# Insulins for the long term management of diabetes mellitus in dogs: a review

**DOI:** 10.1186/s40575-022-00114-9

**Published:** 2022-02-14

**Authors:** Robert E. Shiel, Carmel T. Mooney

**Affiliations:** School of Veterinary Medicine, University College Dublin, Belfield, Dublin 4, Ireland

**Keywords:** Insulin, Dogs, Diabetes mellitus, Pharmacology

## Abstract

The year 2021 marked the centenary of the isolation of a therapeutic form of insulin and its successful use in dogs. This was a landmark moment that subsequently and rapidly led to the commercial manufacture of insulin for use in humans. The impact of insulin was almost miraculous as those destined to die from their diabetes mellitus returned to life. Over the past 100 years, insulin formulations have been modified to attempt to provide a predictable and prolonged duration of action while avoiding the development of hypoglycaemia. This has led to an ever-growing variety of insulin types in human medicine, many of which have subsequently been used in dogs. The purpose of this review article is to provide an overview of available insulin types and their application to the chronic management of canine diabetes mellitus.

## Plain english summary

Diabetes mellitus is a common hormonal disease of dogs. Lifelong insulin therapy is required to prevent death. A small number of insulin preparations are licensed for use in dogs. However, a much larger range of products are available in human medicine. These are also commonly used in dogs, particularly when control cannot be achieved using licensed preparations.

The dog played a pivotal role in early diabetes mellitus research. One hundred years have passed since pancreatic insulin extracts were first used to manage diabetes mellitus successfully in a dog for 76 days. This rapidly led to the widespread availability of similar extracts of bovine or porcine origin in human medicine. However, early use of insulin extracts was hampered by short duration of action. Over the following decades, a variety of modifications were made to these preparations to encourage the slow release of insulin from the site of injection. Addition of the peptide protamine, zinc or a combination of both increased the duration of action of action, allowing for less frequent injections, thereby dramatically improving the welfare of patients. These insulins were known as Neutral Protamine Hagedorn (NPH), lente and protamine zinc insulin (PZI), respectively. Porcine lente and human PZIs are licensed for use in dogs.

Human insulin products became available in the 1980s. Over the last 25 years, a variety of analogue insulin varieties have been developed and used in human diabetic patients. These genetically modified insulin molecules are designed to alter absorption, distribution, metabolism and excretion of the hormone to improve glycaemic control further. Examples include the long acting insulin formulations glargine, detemir and degludec. Insulin glargine forms crystals when the pH of the solution increases following injection. Insulin detemir and degludec form complexes at the injection site, thereby slowing absorption, and also reversibly bind to albumin both in subcutaneous tissues and in circulation. Understanding the mode of actions of these insulins is essential for their safe use in dogs.

## Insulin: a brief history

The dog played a pivotal role in early diabetes mellitus research [1, 2]. The pancreas was first linked to the development of diabetes mellitus in the late nineteenth century when pancreatectomy, performed to determine the role of the pancreas in the digestion and absorption of fats, was unexpectedly shown to result in polyuria and glucosuria in dogs [3]. Several attempts were made over the following 30 years to treat diabetic animals using a variety of pancreatic extracts [2]. Although glucose-lowering effects were demonstrated, these extracts lacked sufficient concentration and purity to allow therapeutic application. This changed in 1921 when Banting and Best determined a method to concentrate the insulin component of pancreatic extract by performing pancreatic duct ligation, which induced atrophy of exocrine tissue, while sparing the islets of Langerhans [4]. The subsequent extract proved successful for the treatment of experimentally induced diabetes mellitus in a dog for 76 days [5]. The response in this dog, named Marjorie, sparked a surge in insulin research and commercialisation. The extraction and purification technique was refined by Collip based on an alcoholic acid extract from bovine and porcine pancreata, which ultimately resulted in the development of a product suitable for use in humans. By 1923, commercial insulin production had commenced in several countries and Banting and his supervisor MacLeod were awarded the Nobel Prize in Physiology or Medicine in the same year.

Early use of insulin was hampered by short duration of action. In the 1930s, this duration was extended by combining the hormone with the basic protein protamine. Mixtures of insulin, protein and zinc were soon available, with the development of protamine zinc insulin (PZI) in the late 1930s and crystalline Neutral Protamine Hagedorn (NPH) insulin in the 1940s. Protamine-free, zinc-based amorphous lente insulins were developed in the 1950s.

Animal based insulins were the mainstay of therapy for approximately 60 years. Genetically engineered, synthetic human insulins were created in the 1970s, with the first recombinant DNA human insulin administered to non-diabetic volunteers in 1980. Over the last 25 years, a variety of analogue insulin varieties have been developed and used in diabetic patients. These genetically modified insulin molecules are designed to alter absorption, distribution, metabolism and excretion of the hormone to improve glycaemic control.

## The structure of insulin

**Fig. 1:**
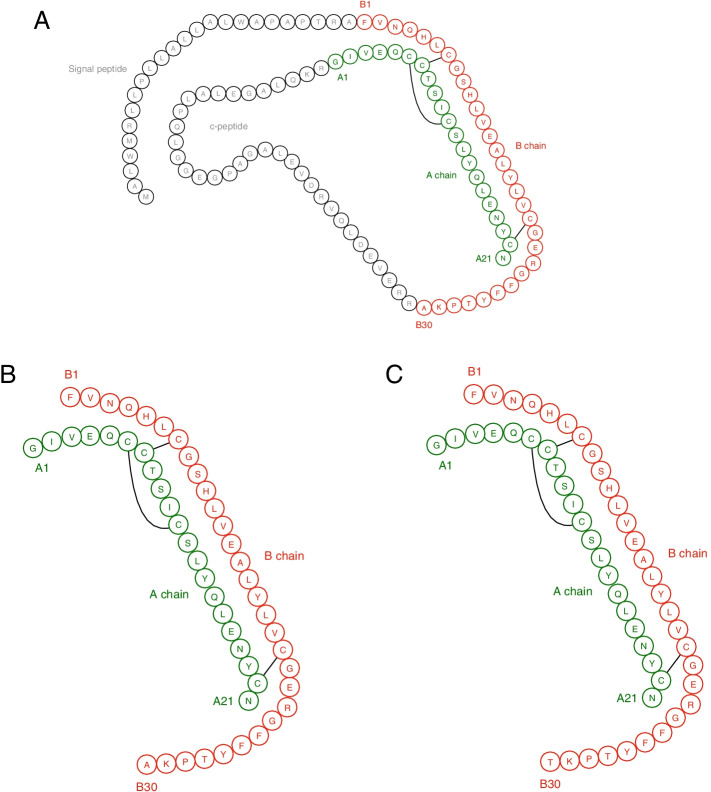
**A **The sequence of the canine pre-proinsulin (predicted from ensembl.org; transcript: ENSCAFT00000016041.5). **B **Cleavage of the signal and c-peptides results in the formation of the mature insulin peptide. **C **The sequence of human insulin is identical to canine insulin except for a single amino acid difference at position B30 (threonine (T) replacing alanine (A)). The black lines represent the position of the disulphide bonds. Note: The figure does not accurately represent the tertiary structure of insulin which is complex and variable

The canine *INS* gene (Gene ID 483665) encodes the 110 amino acid protein preproinsulin. Following cleavage of the signal peptide, proinsulin undergoes folding with the formation of three disulphide bonds to attain its mature three-dimensional globoid conformation. Cleavage of the central c-peptide results in formation of the mature peptide hormone which contains 51 amino acid residues, organised into two polypeptide chains containing 21 and 30 amino acids (A and B chains, respectively) linked by two disulphide bonds (Fig. 1).

**Fig. 2:**
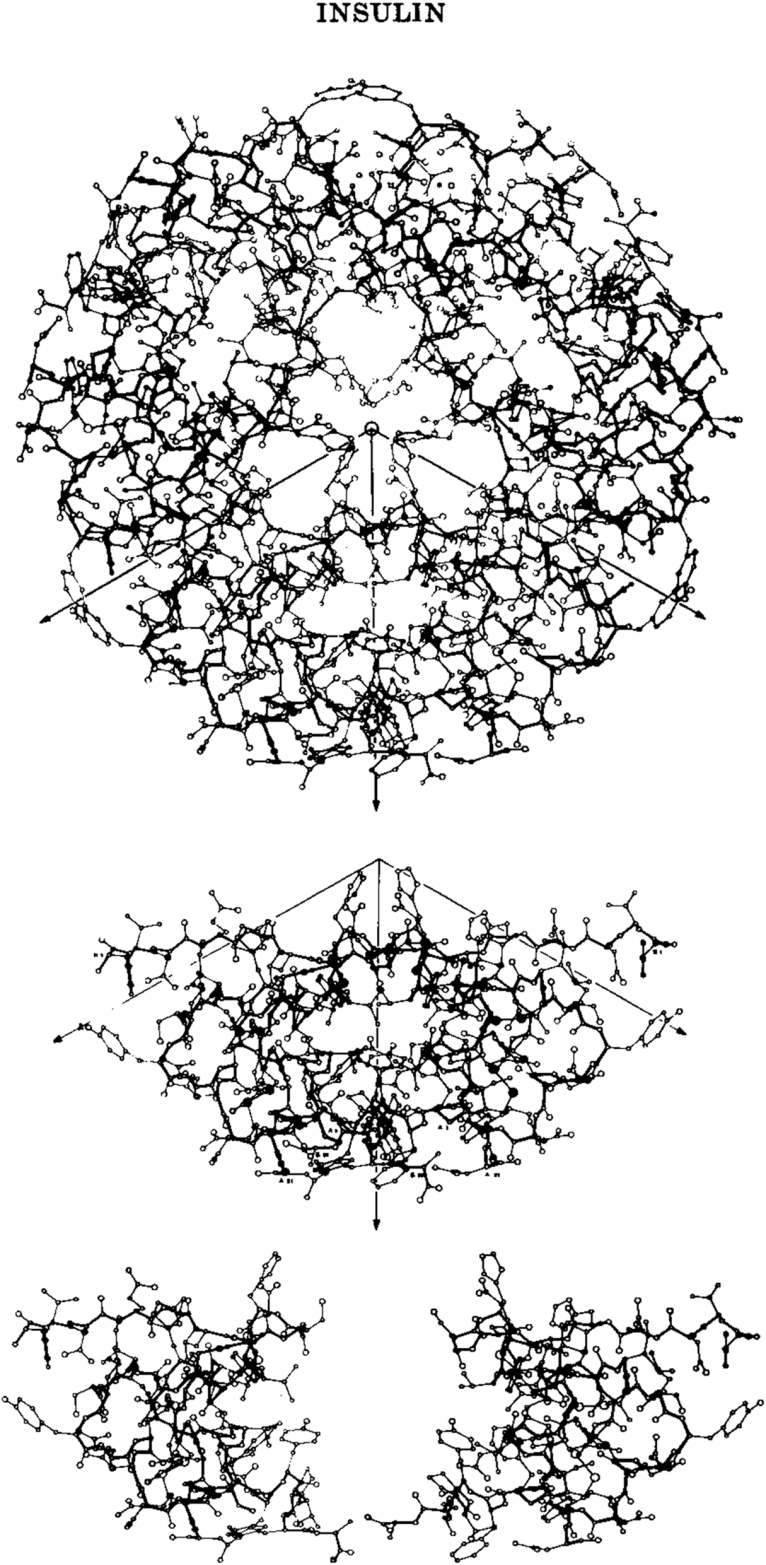
"A thing of beauty is a joy forever". The basic structure of the insulin monomer, dimer and hexamer. From: Blundell et al. 1972 [49]

In solution, insulin undergoes self-assembly into homo-oligomers (i.e. molecules consisting of a small number of identical subunits) in a process depending on temperature, pH, concentration, ionic strength, and the presence of metal ions and other additives [6]. Interaction between the B chains of adjacent monomers results in the formation of dimers. Dimers can be further organised into hexameric structures which are stabilised by divalent ions such as zinc; a histidine residue from each of the constituent B chains are coordinated to two zinc ions resulting in the formation of a symmetric torus-shaped hexamer (Fig. 2).

**Fig. 3:**
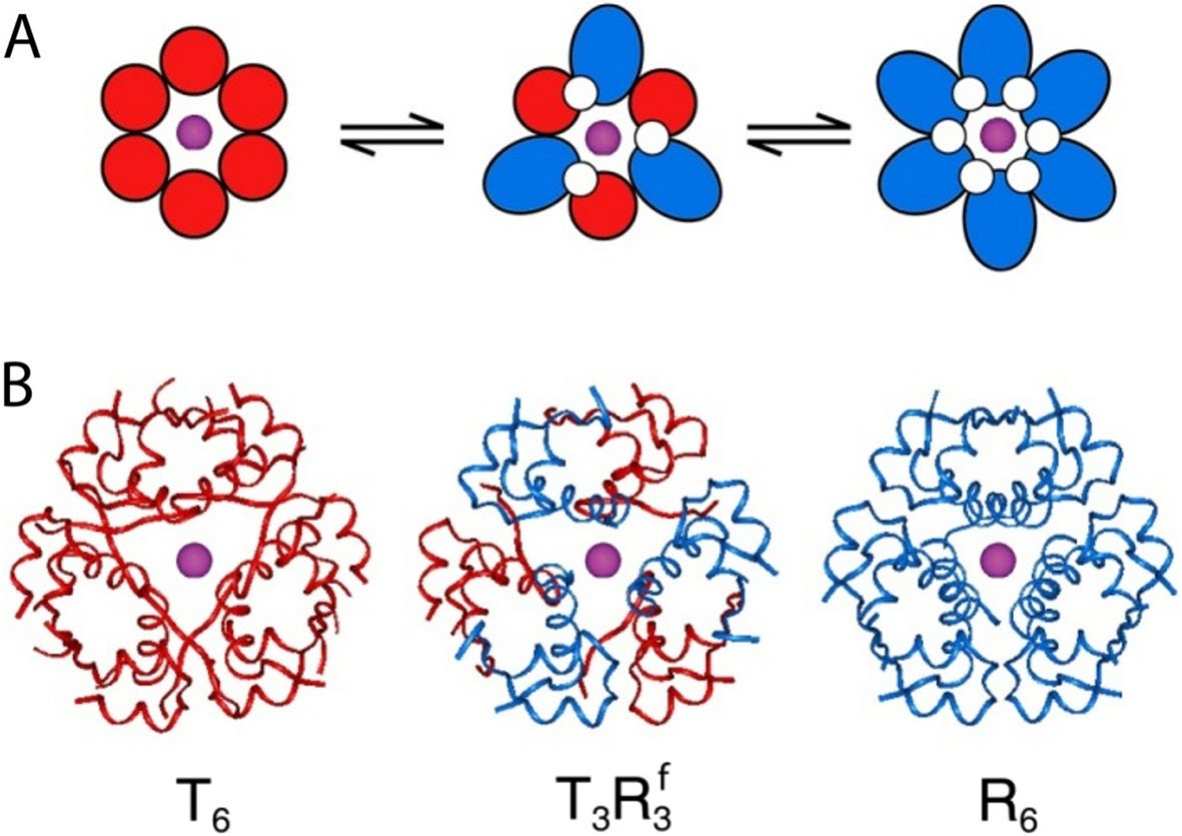
The TR transition between insulin hexamers. **A **Schematic representation of the three insulin hexamers. T state protomers are shown in red, and R state protomers in blue. Axial zinc ions are shown in purple. **B **Corresponding ribbon representation of the crystal structures. From Berenson et al. 2011 [8]

While the monomer is the biologically active form of insulin, hexamers act as a store of pre-formed hormone that is resistant to formation of higher-order aggregates of decreased bioavailability [7]. The classical 2-Zn insulin hexamer is known as T_6_, in which each of the six protomers is in the T state, with a structure similar to that of monomeric insulin [8]. However, three structural families of insulin hexamers are recognised: T_6_, T_3_R^f^_3_ and R_6_ (Fig. 3). Structural rearrangements between the families is termed TR transition. The equilibrium between these forms is determined by salt concentrations and the presence of phenolic ligands. High concentrations of sodium chloride promote formation of T_3_R^f^_3_ hexamers, while phenolic ligands, originally added due to their bacteriostatic properties, induce further transformation to the R_6_ state. These transitions profoundly slow the rate of loss of subunits from the hexamers, reducing the rate of physical and chemical degradation of the peptide chains. These properties are manipulated in several insulin formulations. For example, insulin lispro and insulin aspart form T_3_R^f^_3_ and R_6_ hexamers, respectively, in the presence of phenolic ligands. Upon injection, these ligands rapidly dissociate from the molecule causing reversion to the T6 state. The T6 hexamer rapidly disassembles due to the sequence of these analogues, causing rapid absorption from the injection site.

## Sequence variation between species

Canine and human insulin are identical other than the substitution of alanine by threonine at position B30 (Table 1). Canine and porcine insulin have identical sequences. Bovine insulin differs from canine insulin by substitution of threonine with alanine at A8 and isoleucine with valine at A10.

**Table 1:** Sequence variations between canine, porcine, bovine and human insulin. ALA: alanine; ILE: isoleucine; THR: threonine; VAL: valine

**Species**	**Amino acid position**		
	A8	A10	B30
** Human**	THR	ILE	THR
** Canine/porcine**	THR	ILE	ALA
** Bovine**	ALA	VAL	ALA

In theory, administration of any heterologous peptide could result in antigenic stimulation and an immune response. Weekly administration of bovine but not porcine insulin to dogs in combination with complete Freund adjuvant resulted in anti-insulin antibody production within three weeks of administration [9]. Bovine insulin given in the absence of adjuvant did not result in the development of antibodies within the initial 12 week study period; however, a slight increase in antibody production was noted in several dogs after 10 months of administration.

The increased likelihood of antibody formation to bovine insulin has also been demonstrated in diabetic dogs. The insulin B-chain is most antigenic, although some dogs display reactivity to the whole protein rather than individual subunits [10]. Over 50% of dogs treated with bovine insulin are anti-insulin antibody positive compared to only 12.6% of dogs treated with porcine insulin [11, 12]. The presence of autoantibodies in dogs treated with homologous insulin could reflect the development of naturally occurring autoantibodies as part of type 1 diabetes. Indeed, a similar proportion of anti-insulin antibodies has been described in diabetic dogs prior to commencing therapy [11]. The generation of this response appears to be breed dependent, with the dachshund, Cairn terrier, miniature schnauzer and Tibetan terrier more likely, and the cocker spaniel less likely, to develop anti-insulin antibodies [12]. Furthermore, the dog leukocyte antigen (DLA) haplotype DRB1*0015/DQA1*006/DQB1*023 was positively associated, and DLA-DRB1*006/DQA1*005/DQB1*007 negatively associated, with the development of anti-insulin autoantibodies. The clinical significance of anti-insulin antibodies is unknown, but it has been suggested to contribute to lack of efficacy of insulin at least in some cases. This may not be immediately reversible following a change of insulin type because antibodies formed by exposure to heterologous insulin cross react with homologous insulin [10].

## Types of insulin used for the chronic management of canine diabetes mellitus

**Fig. 4:**
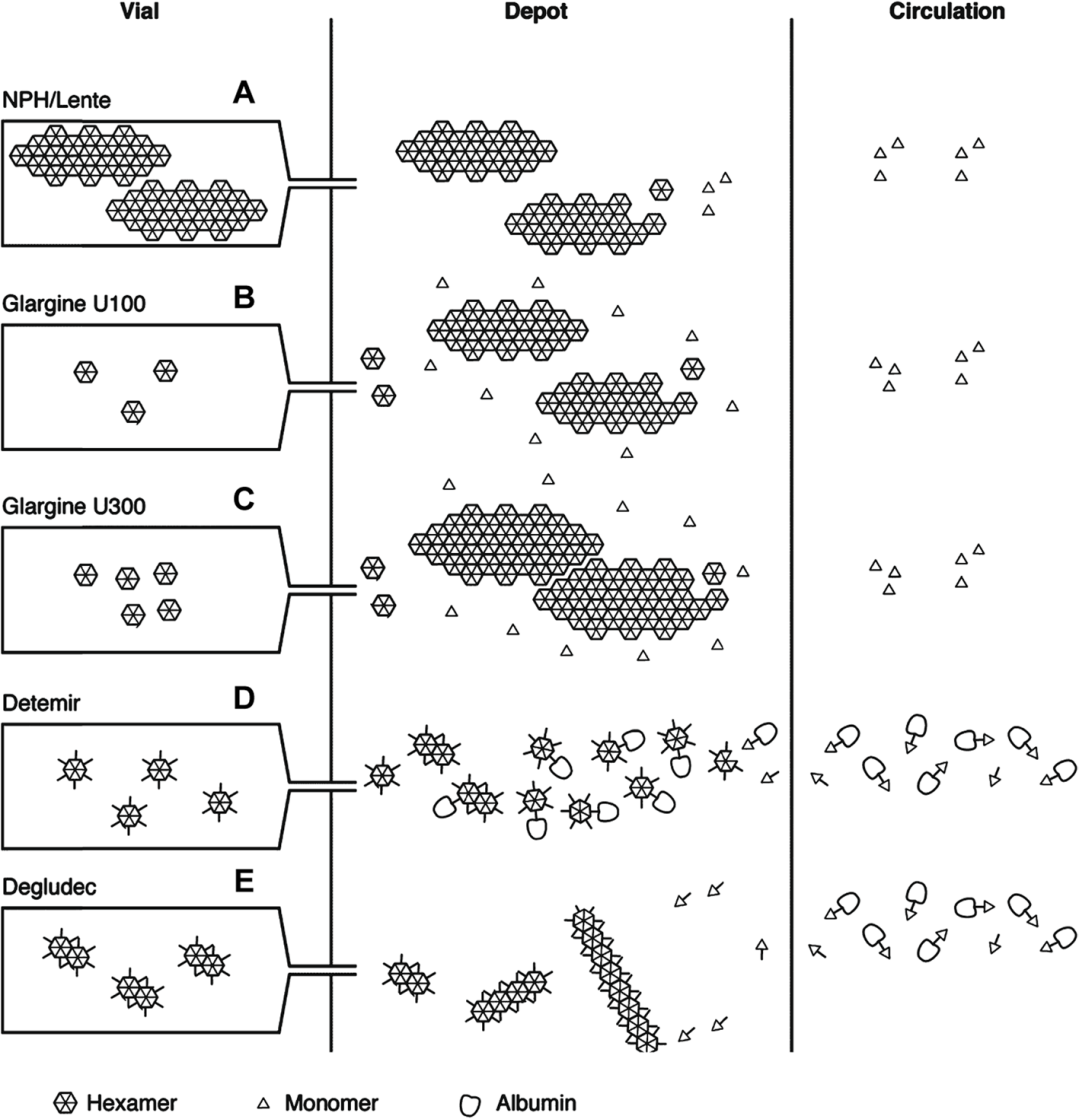
Summary of the mechanism of protraction of insulin action. Insulins vary in the size and type of crystal formed at the site of injection, and the ability to interact with albumin. NPH insulin (**A**) is injected as a pre‐formed protein–insulin conglomerate. Insulin glargine (100 U/mL) (**B**) forms crystals when the pH increases following injection. Insulin glargine 300 U/mL (**C**) also precipitates at physiological pH but these precipitates are more compact compared with the 100 U/mL preparation, reducing the surface area for absorption. Insulin detemir (**D**) hexamers self-associate at the injection site into dihexamers, thereby slowing absorption, and reversibly bind to albumin both in subcutaneous tissues and in circulation. Insulin degludec (**E**) has similar properties, but further protraction of absorption is achieved by multihexamer chain formation at the site of injection. Subsequent dissociation of zinc causes the terminal hexamers to break down. Modified from Heise and Mathieu, 2017 [25]

A variety of insulin types have been used in the chronic management of canine diabetes mellitus. In general, these products are used as background or basal insulins to provide a long-acting background concentration of the hormone in the body throughout the day, avoiding extremes of hypoglycaemia and prolonged or severe hyperglycaemia. These insulins have a protracted duration of activity, due to altered interaction between monomers or other proteins such as albumin. This is achieved by exploiting the factors influencing native insulin crystal formation or modifying the insulin sequence to create analogues (Fig. 4). These insulins are classified as intermediate or long-acting, although there may be overlap in duration of action between these two groups in individual patients.

Regular insulin or rapid acting analogue bolus insulins (e.g. lispro, aspart or glulisine) are used in people for control of blood glucose concentration at meal times or other periods of hyperglycaemia. Such insulins are rarely used in dogs other than the acute management of severe forms of diabetes mellitus such as diabetic ketoacidosis or the hyperosmolar, hyperglycaemic state.

Most insulins are 100 U/mL, other than the licensed veterinary lente and protamine zinc insulins, which are 40 U/mL, and certain analogue insulins of higher concentration (e.g. glargine 300 U/mL or degludec 200 U/mL).

Comparison between the studies assessing performance of insulin formulations in dogs is difficult for several reasons. Different brands of the same type of insulin may not behave in an identical way [13]. Many studies are small, often with ten or fewer cases. Therefore, results from individual dogs can dramatically skew results. Some reports use dogs with experimentally induced diabetes mellitus in a research setting, while others describe the response to treatment of client-owned dogs with naturally occurring diabetes mellitus. The administration of insulin and food by owners adds huge variability, but also reflects clinical practice more accurately. Signalment and inclusion and exclusion criteria vary, and in some cases, the extent of investigation of concurrent disease is unclear. Treatment trials are often of different duration, and determination of success may be based on improvement in clinical signs, clinicopathological features (blood glucose or serum fructosamine measurement), or both, with inconsistent targets for good, moderate and poor control. Duration of action may be classified using the duration blood glucose concentrations are below the baseline value or a variable percentage of the baseline value, the duration insulin is detected in blood, or glucose infusion rates under euglycemic glucose clamp conditions. Diets are not always standardised, and vary between studies. As a result, differences between the results from individual studies must be interpreted with some caution.

### Intermediate-acting insulins

#### Lente insulin

Lente insulins are protamine-free preparations, made by complexing neutral suspensions of insulin with small quantities of zinc to produce crystals of varying size. Larger crystals are associated with slower dissolution from the subcutaneous depot and prolonged duration of action. Three types of lente insulin are described: semilente, ultralente and lente. Amorphous or semilente insulin contains small crystals with a short duration of action. Microcrystalline or “ultralente” insulin contains larger crystals and has a longer duration of action. Lente insulin is made by mixing semilente and ultralente formulations, typically in a ratio of approximately 3:7 and is considered to be intermediate acting.

The physical state and size of the insulin crystals can be manipulated by altering the pH and zinc content of the preparation [14]. For ultralente insulin, initial T_3_R^f^_3_ crystals are grown at pH 5.5 before transition to a pH of 7.4 in the presence of zinc ions and sodium chloride, which results in transition to T6 crystals. By contrast, semilente precipitates are produced in a one stage process at pH 7.4. Further stability is provided in the presence of methylparaben, which also acts as an antimicrobial preservative.

Bovine and porcine lente insulins were first developed in the 1950s and were widely used in human medicine for several decades. Human insulins became available in the 1980s, initially derived semi-synthetically by enzymatic modification of porcine insulin, and later using recombinant protein technology [15]. Although formulations of human lente insulin were developed, these products had a shorter duration of action, particularly compared to the bovine formulation [15]. This may reflect lower solubility of bovine insulin at a neutral pH [16]. The reduced duration of glycaemic control, combined with the variability of action due to inconsistent resuspension, the more widespread use of NPH insulin, and the later development of more reliable long-lasting human analogue insulins, led to the discontinuation of human lente insulins in the mid 2000s.

Porcine lente insulin is the most commonly used preparation for chronic management of canine diabetes mellitus. Two licenced veterinary formulations are produced by the same parent company, but marketed under different tradenames in different countries: Caninsulin (MSD Animal Health) and Vetsulin (Merck Animal Health). Both formulations contain porcine insulin at a concentration of 40 U/mL. The ratio of semilente to ultralente insulin is 3:7 and 35:65, respectively. In dogs, administration of lente insulin results in either one or two peaks of activity [17]. The first, higher peak occurs approximately 4.3 h after subcutaneous injection. The second, lower peak occurrs after approximately 11 h, but is not seen in all dogs. In practice, this results in a nadir blood glucose concentration 4–8 h after insulin administration, and a reduction in glucose concentration ranging from 8–24 h [16, 17].

In a longer term study of 53 dogs, the mean blood glucose concentration was 10.2 mmol/L, and the mean nadir value was 6.6 mmol/L after 60 days of therapy following completion of an initial dose determination period [18]. Adequate glycaemic control was achieved in 75% of cases, defined as adequate control of polyuria, polydipsia, polyphagia and ketonuria, cessation of weight loss and glucose concentrations < 13.9 mmol/L for 16 or more hours per day. All dogs in this study commenced with once daily insulin, but two thirds required transition to twice daily therapy due to persistence of clinical signs and failure to maintain blood glucose concentrations below 13.9 mmol/L for 12– 16 h during a blood glucose curve. Although the manufacturer of the licensed formulations advises initial once daily administration, this preference for twice daily therapy to achieve optimal glycaemic control has been reported in several studies and the majority of clinicians standardly use twice daily therapy from the outset [18–21]. However, this could result in an increased risk of hypoglycaemia in some dogs with a duration of insulin action > 12 h [22].

#### Neutral Protamine Hagedorn Insulin

Neutral Protamine Hagedorn insulin is an intermediate-acting insoluble insulin made by co-crystallisation of the hormone with zinc in the presence of the basic peptide protamine, originally derived from fish sperm [23]. This process of combining protamine and insulin was first described by the Danish clinician Hans Christian Hagedorn in the 1930s [24]. However, the original neutral suspension was chemically unstable; a problem overcome 10 years later when insulin and protamine were combined in isophane proportions (i.e. no excess of protamine or insulin, at a ratio of 5:1) at a neutral pH in the presence of zinc and phenols to produce a suspension of crystalline NPH insulin. During subcutaneous administration, crystals are laid in “heaps” at the injection site, from which the hormone is gradually released into circulation. The structure of the insulin protamine complexes and method of dissociation are not fully known. However, the process involves insulin release following diffusion of phenolic preservatives from the injection site, with further crystal dissolution driven by protamine splitting enzymes and macrophages [25]. Although insulin release is protracted, it occurs at a variable speed due to a lack of consistency of the size and shape of the insulin-protamine complexes, the injection site environment, and differing degrees of resuspension with mixing, resulting in a lack of consistency between injections.

Neutral Protamine Hagedorn insulin was the most widely used insulin in canine medicine, particularly prior to the availability of a licensed lente formulation [16, 21, 26]. Early studies described bovine NPH formulations, but only human recombinant preparations are currently available [16]. Comparison between studies is further complicated by manufacturing differences between biosimilar products [13]. The duration of action of a human recombinant NPH, defined as the length of time a blood glucose concentration remained below the pre-injection value, ranged from 4–10 h in 10 diabetic dogs [27]. The nadir occurred an average of 4.9 h after insulin administration, but ranged from 1–10 h. As a result, twice daily administration is recommended [21, 27, 28]. Results of a recent prospective randomised study of 30 newly diagnosed diabetic dogs suggested that lente and human recombinant NPH insulins were similarly effective for the management of canine diabetes mellitus [29]. Although the study was small, this suggests NPH insulin may be used to control diabetes mellitus in dogs successfully.

Postprandial hyperglycaemia has been described in five of 10 dogs receiving NPH insulin [27]. This led to the suggestion that NPH combined with short acting insulin, such as regular insulin, could help eliminate this postprandial blood glucose spike and improve glycaemic control [21]. Recently, a combination of lispro and NPH insulins in six diabetic dogs, was shown to significantly lower serum fructosamine and postprandial blood glucose concentrations in diabetic dogs [30].

### Long-acting insulins

#### Protamine zinc insulin

Protamine zinc insulin (PZI) is created using excess protamine and a small quantity of zinc, which results in a suspension with a prolonged hypoglycaemic effect of up to 24 h in humans but a slow onset of action.

In dogs, PZI has been described for the management of diabetes mellitus and a licensed recombinant human insulin formulation is available for use in dogs containing 40 U/mL (Prozinc, Boehringer Ingelheim). The manufacturer recommends commencing with once daily therapy. In 10 healthy dogs, the median onset of action was 3.5 h (range 0.5—10 h) and the nadir occurred after a median of 14 h (range 5—> 24 h) [31]. The median duration of action was > 24 h (range 16—> 24 h), calculated as the time taken for insulin to return to a value not significantly lower than the basal concentration. This variability in absorption and action had been previously described using bovine PZI in diabetic dogs [16]. This is not unique to PZI, and has also been described when using both NPH and lente formulations, but the marked variability of PZI may make the glycaemic response less predictable [16]. In diabetic dogs, nadir values were observed to occur between two and 12 h after administration [32]. This insulin can be used to control diabetes mellitus effectively in dogs, including some dogs in which lente insulin was not successful, presumably reflecting the longer duration of action [32]. In the largest study to date, including dogs on both once and twice daily recombinant human PZI, 41/220 (18.6%) dogs had good glycaemic control when defined as a mean blood glucose concentration during a blood glucose curve ≤ 11.1 mmol/L, and 113 (51.4%) when defined as a fructosamine concentration ≤ 450 umol/L, by day 84 of treatment [33]. Nadir blood glucose values were < 8.3 mmol/L in 89 (40.5%) dogs. After six months, 107/187 (57.2%) were still receiving once daily therapy. The remaining dogs were placed on twice daily therapy due to an inadequate response on once daily therapy and an inability to increase the dose because of concerns for hypoglycaemia.

#### Analogue insulins

Analogue insulins are genetically engineered to overcome some of the limitations of NPH and lente insulin such as variable absorption, duration of effect and risk of hypoglycaemia. These have become more popular over the last 25 years in human medicine and their use in canine practice has also grown. These insulins are also solutions rather than suspensions, which decreases variability between injections from incomplete mixing prior to administration.

##### Insulin glargine

Insulin glargine was the first insulin analogue approved for use in humans in the year 2000, produced by substituting glycine for asparagine at position A21, and adding two asparagine molecules to the carboxy terminal of the B chain. Insulin glargine has an isoelectric point at pH 6.7, compared to 5.4 for the native molecule [34]. This results in decreased solubility at a neutral pH. After injection, the acid in the vehicle is neutralised and the insulin precipitates in subcutaneous tissues from where it is gradually released into circulation.

Insulin glargine was first produced at a concentration of 100 U/mL. A more concentrated formulation of 300 U/mL was subsequently developed which resulted in a more compact subcutaneous depot and a slower and more prolonged release of insulin [35]. At steady state, the 300 and 100 U/mL formulations have a half lives of 19 and 13.5 h in humans, respectively. It became the most widely used insulin due to its long duration of action, lack of pronounced peak activity, and lower frequency of hypoglycaemic events [36]. However, glargine did not control postprandial hyperglycaemia, making it necessary to supplement with short-acting insulin at mealtimes.

The time-action profiles of glargine 100 U/mL and NPH insulins have been compared in three healthy dogs and three dogs with experimentally induced diabetes [37]. In healthy dogs, peak activity was detected at 5 h for NPH and 7 h for glargine insulin, with a duration of effect of 12 and 24 h, respectively. The NPH insulin was more effective than glargine during the first six hours following administration. Although only three healthy dogs were included, the time-pattern profile curve was more consistent for NPH compared to insulin glargine, suggesting greater variability between individual dogs receiving insulin glargine. Administration of twice daily glargine has also been described in 12 diabetic dogs that were fed a balanced diet without simple sugars [38]. After 24 weeks of therapy, the average blood glucose concentration during blood glucose curves was 12.4 mmol/L (range 5.2–17.6) with an average nadir value of 7.0 mmol/L (range 3.3–12.3). Nadir values were most commonly observed 6–8 h after insulin administration, but could occur at any time of day. Good glycaemic control was achieved in 7/12 dogs, defined as good clinical control and mean blood glucose concentrations during blood glucose curves of < 13 mmol/L. By contrast, a later studied evaluated the efficacy of insulin glargine in 10 diabetic dogs fed a standardised high-fibre diet [39]. There was no significant difference between mean minimum and maximum blood glucose concentrations within the glucose curves, which suggested that glargine was a peakless insulin when using this protocol, although the power of the study was likely low. All dogs were described to have well-regulated diabetes mellitus prior to or at the completion of the study. However, dogs were enrolled in the study for a relatively short period (36–78 days), and dogs were considered to be well-regulated based on owner reported clinical signs.

Few studies have assessed the use of 300 U/mL insulin glargine in dogs. In eight healthy dogs, the median time to onset of action was 4 h (range 3.3–5.8 h), median time to peak action was 6.3 h (range 5.0–21.3 h) and median duration of action was 16.3 h (range 6.1 to 20.1 h) [40]. This is not dissimilar to the values described above for 100 U/mL glargine [37]. However, this may have reflected methodological differences between the studies. In a separate study which directly compared the 300 and 100 U/mL formulations in healthy dogs, the former was associated with a slower and less marked decrease in glucose concentration, and mean residency time of the analogue insulin was 32% longer, and bioavailability was 50% lower compared to the latter formulation [41]. This suggests that the higher concentration formulation may have a more protracted duration of action, and may be a better choice for once daily insulin therapy. Decreased day-to-day variability, determined as a lower mean coefficient of variation over 5 days of therapy, has also been shown for this formulation of insulin glargine when administered twice daily compared to porcine lente insulin [42]. This could suggest decreased monitoring requirements in dogs receiving this insulin type twice daily. However, intra-day variability, measured by the glycaemic variability percentage, was higher for porcine lente compared to glargine 300 U/mL insulin.

##### Insulin detemir

Insulin detemir is a pH neutral soluble insulin analogue that is acylated at position B29 with a 14-carbon fatty acid residue. This modification increases self-association of the molecules at the subcutaneous injection site as dihexamers, and allows reversible binding to albumin, slowing its absorption.

The time action profiles of insulin detemir have been described in three healthy and eight diabetic dogs [43]. In healthy dogs, a pronounced insulin peak was identified 8–10 h post administration, with an insulin effect that was persistent for over 24 h. However, in a separate study of eight healthy dogs, the median time-to-peak and median duration of action of insulin detemir (4.3 (range 2.9–7.4) and 10.8 (range 8.8–14.8) hours, respectively) was not significantly different to glargine (6.3 (range 5.0–21.3) and 16.3 (range 6.1–20.1) hours, respectively) [40]. The time to onset of action of insulin detemir is short, with a median of 0.6 h (range 0.6–1.2 h) [40]. In diabetic dogs, the mean blood glucose concentration was lower with less variability in dogs receiving insulin detemir compared to glargine and NPH insulins [43]. Lower doses of detemir are required to maintain glycaemic control in dogs; therefore, care should be taken to commence therapy using a lower dose compared to other insulin types [21, 44]. In addition, accurate dosing may not be possible in smaller dogs weighing less than 10–15 kg. Good glycaemic control, based upon control of clinical signs and a median blood glucose concentration in the blood glucose curves < 13 mmol/L, was attained in 5/10 dogs after 24 weeks [44].

##### Insulin degludec

Insulin degludec is modified by the deletion of threonine at position B30 and the addition, by a glutamic acid linker, of a 16-carbon fatty diacid to lysine at position B29 [45]. This fatty acid group allows the insulin molecules to self-associate, in the presence of zinc and phenol, to form dihexamers, containing 12 insulin monomers. Following depletion of phenol, these dihexamers further organise to form linear multihexamers that precipitate at the injection site. Gradual diffusion of zinc from the injection site allows slow release of insulin monomers from this depot, which are subsequently absorbed into circulation. The acylation at B29 also allows the molecule to bind to albumin in the bloodstream, prolonging its action in a mechanism similar to detemir.

Time action profiles for insulin degludec 100 U/mL have also been reported in five healthy and four diabetic dogs [46]. In healthy dogs, an indistinct peak of activity was observed after 9–13.5 h. The a duration of action > 20 h, similar to that described above in insulin glargine and insulin detemir in similar studies at the same institution [37, 43]. In diabetic dogs, there was little significant difference between the success of control of diabetes when compared to recombinant human NPH insulin in the same groups of dogs [46]. However, the study was small, containing only four dogs. It was also suggested that glycaemic control could be complicated by higher postprandial and lower preprandial blood glucose concentrations associated with insulin degludec use. By contrast, similar to glargine 300 U/mL, decreased day-to-day variability, determined as a lower mean coefficient of variation over 5 days of therapy, has also been shown for insulin degludec 100 U/mL when administered twice daily compared to porcine lente insulin [42]. No significant differences in day-to-day or inter-day variability were identified between insulin glargine 300 U/mL and insulin degludec 100 U/mL.

### Ultra-long acting insulin formulations

A single abstract has described the use of a novel once weekly recombinant canine insulin in four diabetic dogs following transition from standard twice daily insulin therapy [47]. Although the study was small, there were no significant differences in the severity of clinical signs, body weight, fructosamine concentration, or mean interstitial glucose concentrations after 8 weeks of treatment. No adverse effects were reported. The development of such a potentially long-acting insulin could revolutionise management of canine diabetes mellitus, especially given the perceived impact of the need to administer at least daily insulin injections on the quality of life of diabetic dogs and their owners [48].

## Conclusions

A wide array of insulin types are available for the chronic management of diabetes mellitus in dogs, which vary in potency, speed of absorption and duration of action. Most animals can be stabilised using the licensed veterinary formulations. However, in dogs that are challenging to control, alternative insulins may be more appropriate, particularly when instability is due to short or long duration of action in an individual animal. Knowledge of the behaviour of these insulins is essential to use them effectively and avoid adverse effects.

## Data Availability

not applicable.

## Abbreviations

NPH: Neutral Protamine Hagedorn
PZI: Protamine Zinc Insulin
U: Units
