# Correction to: Insulins for the long term management of diabetes mellitus in dogs: a review

**DOI:** 10.1186/s40575-022-00115-8

**Published:** 2022-03-04

**Authors:** Robert E. Shiel, Carmel T. Mooney

**Affiliations:** School of Veterinary Medicine, University College Dublin, Belfeld, Dublin 4, Ireland

**Correction to: Canine Med Genet 9, 1 (2022)**

**https://doi.org/10.1186/s40575-022-00114-9**

Following publication of the original article [1], it was noted that due to a typesetting error Figure 2’s caption was not complete, and Fig. 4 was not correct. Figure 2’s caption should be:

**Fig 2.** “A thing of beauty is a joy forever”. The basic structure of the insulin monomer, dimer and hexamer. From: Blundell et al. 1972 [49].

In addition, the correct Fig. 4 is given below:

**Fig. 4:**
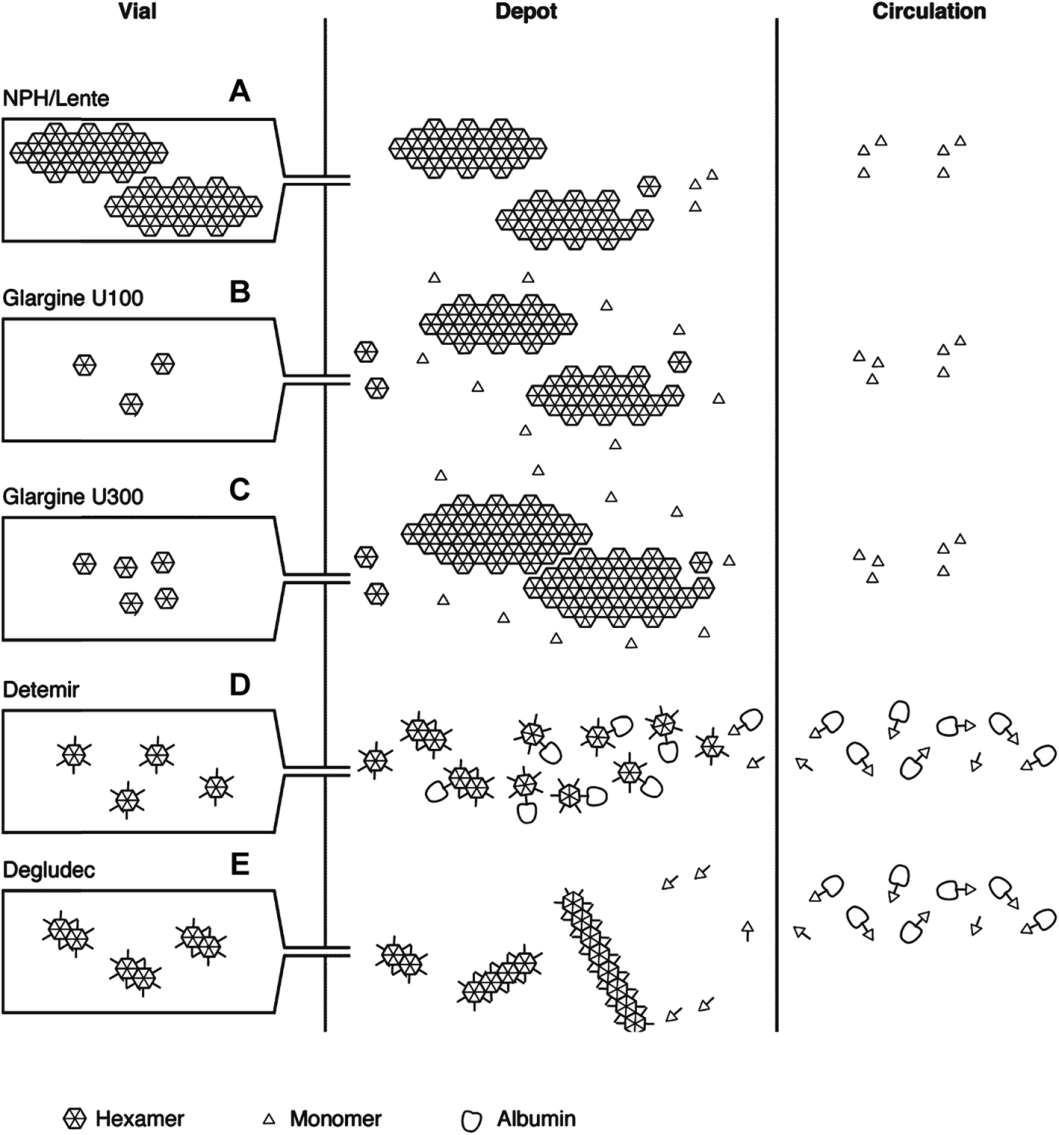
Summary of the mechanism of protraction of insulin action. Insulins vary in the size and type of crystal formed at the site of injection, and the ability to interact with albumin. NPH insulin (**A**) is injected as a pre‐formed protein–insulin conglomerate. Insulin glargine (100 U/mL) (**B**) forms crystals when the pH increases following injection. Insulin glargine 300 U/mL (**C**) also precipitates at physiological pH but these precipitates are more compact compared with the 100 U/mL preparation, reducing the surface area for absorption. Insulin detemir (**D**) hexamers self-associate at the injection site into dihexamers, thereby slowing absorption, and reversibly bind to albumin both in subcutaneous tissues and in circulation. Insulin degludec (**E**) has similar properties, but further protraction of absorption is achieved by multihexamer chain formation at the site of injection. Subsequent dissociation of zinc causes the terminal hexamers to break down. Modified from Heise and Mathieu, 2017 [25]

The original article has been corrected.
